# The Impact of Non-Invasive Scores and Hemogram-Derived Ratios in Differentiating Chronic Liver Disease from Cirrhosis

**DOI:** 10.3390/jcm14093072

**Published:** 2025-04-29

**Authors:** Abdulrahman Ismaiel, Evrard Katell, Daniel-Corneliu Leucuta, Stefan-Lucian Popa, Cristina Sorina Catana, Dan L. Dumitrascu, Teodora Surdea-Blaga

**Affiliations:** 1 2nd Department of Internal Medicine, “Iuliu Hatieganu” University of Medicine and Pharmacy, 400006 Cluj-Napoca, Romania; abdulrahman.ismaiel@yahoo.com (A.I.); popa.stefan@umfcluj.ro (S.-L.P.); ddumitrascu@umfcluj.ro (D.L.D.); dora_blaga@yahoo.com (T.S.-B.); 2 Faculty of Medicine, “Iuliu Hatieganu” University of Medicine and Pharmacy, 400394 Cluj-Napoca, Romania; 3 Department of Medical Informatics and Biostatistics, “Iuliu Hatieganu” University of Medicine and Pharmacy, 400349 Cluj-Napoca, Romania; dleucuta@umfcluj.ro; 4 Department of Medical Biochemistry, “Iuliu Haţieganu” University of Medicine and Pharmacy, 400012 Cluj-Napoca, Romania; ccatana0128@gmail.com

**Keywords:** chronic liver disease (CLD), cirrhosis, non-invasive biomarkers, fibrosis scoring systems, hemogram-derived ratios

## Abstract

**Background:** Chronic liver disease (CLD) is a major global health concern, contributing significantly to morbidity and mortality. Cirrhosis and liver cancer are the leading causes of liver-related deaths, with various etiological factors, such as metabolic disorders and alcohol-related and viral hepatitis, driving its global prevalence. Non-invasive biomarkers and scoring systems have emerged as key tools for assessing liver disease severity and differentiating CLD from cirrhosis. This study evaluates biomarkers and non-invasive scores and their utility in distinguishing CLD from cirrhosis. **Methods:** This retrospective observational study included 250 adult patients hospitalized between January 2021 and December 2023 at Cluj County Emergency Clinical Hospital, Romania. Patients were diagnosed with either cirrhosis or CLD of viral, autoimmune, or primary biliary cholangitis (PBC) etiology. Non-invasive biomarkers, scores, and various hemogram-derived ratios were evaluated. Statistical analysis involved descriptive statistics, comparative tests, and receiver operating characteristic (ROC) curve analysis. **Results:** Among the 250 patients, 137 had liver cirrhosis (54.8%) and 113 had CLD without cirrhosis (45.2%). Significant differences were observed in laboratory parameters, with cirrhosis patients showing lower hemoglobin, platelet count, and albumin levels alongside higher liver enzymes and INR values. Non-invasive scores such as APRI, FIB-4, and NFS demonstrated higher values in the cirrhosis group, indicating more advanced liver damage. Hemogram-derived ratios, particularly the neutrophil-to-lymphocyte ratio (NLR), were higher in cirrhosis patients. ROC analysis revealed that the Lok index had the highest discriminatory power (AUC 0.89), followed by the King score (AUC 0.864) and the Fibrosis index (AUC 0.856), which effectively distinguished cirrhosis from CLD. **Conclusions:** This study underscores the utility of non-invasive biomarkers and scoring systems in differentiating CLD from cirrhosis. The Lok index, King score, and Fibrosis index demonstrated excellent diagnostic accuracy, while hemogram-derived ratios, such as NLR, offer insights into systemic inflammation associated with liver disease progression. These findings support the integration of non-invasive markers into clinical practice for improved risk stratification and management of liver diseases.

## 1. Introduction

Chronic liver disease (CLD) represents a significant global health burden, contributing to substantial morbidity and mortality [1]. According to the World Health Organization, liver diseases account for approximately 2 million deaths annually, with cirrhosis and liver cancer being the leading causes [2]. The global prevalence of CLD is driven by various etiological factors, including viral hepatitis, autoimmune hepatitis, alcohol consumption, and metabolic disorders [3]. Despite advances in therapeutic strategies, the silent progression of CLD often results in late-stage presentation, underscoring the need for early detection and accurate risk stratification. Timely identification of patients at risk of progressing to cirrhosis is crucial for optimizing management and improving clinical outcomes.

Viral hepatitis, particularly hepatitis B and C, remains a major cause of CLD worldwide, with antiviral therapies playing a pivotal role in disease control. Autoimmune liver diseases, including autoimmune hepatitis (AIH), stem from aberrant immune responses against hepatic tissues, leading to chronic inflammation and fibrosis. Primary biliary cholangitis (PBC), a chronic cholestatic disease, results from the progressive destruction of intrahepatic bile ducts, ultimately causing biliary fibrosis and cirrhosis. The heterogeneity of CLD etiologies necessitates a tailored diagnostic and therapeutic approach [4].

The natural evolution of CLD is characterized by a prolonged asymptomatic phase, during which hepatic fibrosis gradually progresses [5]. Persistent liver injury and inflammation trigger fibrogenesis, culminating in the formation of bridging fibrosis and, ultimately, cirrhosis [6]. Once cirrhosis develops, patients face an elevated risk of complications, such as portal hypertension, variceal bleeding, ascites, hepatic encephalopathy, and hepatocellular carcinoma (HCC) [7]. The transition from compensated to decompensated cirrhosis marks a critical inflection point, significantly worsening the prognosis. Understanding the dynamic progression of CLD is vital for early intervention and reducing the burden of end-stage liver disease.

Non-invasive biomarkers and scoring systems have emerged as essential tools for assessing liver disease severity, predicting disease progression, and differentiating CLD from cirrhosis. Non-invasive markers such as liver stiffness measurements (via transient elastography), serum fibrosis markers (e.g., FIB-4, APRI), and imaging-based indices offer reliable alternatives to liver biopsy [8,9]. Moreover, existing literature lacks side-by-side comparisons of these scores with newer or composite indices, such as the Lok index and King score. Despite the growing body of evidence supporting the use of non-invasive markers in liver disease, there remains a lack of comparative studies specifically evaluating their diagnostic performance across CLD of viral, autoimmune, and cholestatic etiologies. Furthermore, hemogram-derived ratios, although increasingly recognized as markers of systemic inflammation, have not been extensively assessed for their discriminatory value between non-cirrhotic CLD and established cirrhosis in such a targeted population. Hemogram-derived ratios, including the neutrophil-to-lymphocyte ratio (NLR) and platelet-to-lymphocyte ratio (PLR), have shown promise in reflecting underlying inflammation and portal hypertension [10]. The integration of these non-invasive methods into routine clinical practice enhances risk stratification and guides treatment decisions.

The present study aimed to evaluate and compare cirrhosis with CLD of viral, autoimmune, and PBC etiologies, with a particular focus on comparing various biomarkers and non-invasive scores for predicting hepatic steatosis and liver fibrosis. Furthermore, we sought to assess the utility of several non-invasive scores for hepatic steatosis and liver fibrosis, including the Lok index, King score, and Fibrosis index, as well as hemogram-derived ratios, in distinguishing CLD patients from those with liver cirrhosis. Our study addresses this gap by providing a detailed evaluation of both conventional and emerging non-invasive indices. This approach allows for a head-to-head comparison of multiple tools in differentiating CLD from cirrhosis, offering practical insights into their relative strengths and potential for integration into everyday clinical practice, with the potential to optimize risk stratification and minimize the need for invasive diagnostics. By elucidating these diagnostic and prognostic parameters, our study aims to contribute to the growing body of evidence supporting non-invasive strategies for personalized CLD management.

## 2. Methods

### 2.1. Study Participants

This retrospective observational study was conducted at the Cluj County Emergency Clinical Hospital, Cluj-Napoca, Romania, over a three-year period from January 2021 to December 2023. The study population comprised adult patients hospitalized with a confirmed diagnosis of either cirrhosis, irrespective of its etiology, or chronic viral or autoimmune liver disease. The diagnosis of cirrhosis was established based on a comprehensive assessment, integrating clinical findings, biochemical parameters, and imaging techniques, including abdominal ultrasound, transient elastography (FibroScan), computed tomography (CT), magnetic resonance imaging (MRI)—as well as liver biopsy and endoscopic evaluations where available.

To ensure diagnostic consistency, patients with metabolic dysfunction-associated steatotic liver disease (MASLD), alcohol-related liver disease (ALD), or metabolic and alcohol-related liver disease (MetALD) were excluded from the CLD group, as retrospective data did not allow reliable quantification of alcohol intake or assessment of metabolic dysfunction components. However, cirrhosis cases were included regardless of etiology, as cirrhosis represents the final stage of liver disease, where etiologies are typically confirmed through cumulative clinical imaging and biochemical criteria. This approach allowed a focused comparison between clearly characterized non-cirrhotic CLD and established cirrhosis while reflecting the broader etiologic spectrum seen in clinical practice. Each patient was enrolled in the study only once, regardless of the number of hospital admissions during the study period. Eligible participants were identified using specific International Classification of Diseases (ICD) codes: B18.0, B18.1, B18.2, B18.9 for chronic viral hepatitis; K70.3 and K74.6 for liver cirrhosis; K73.0, K73.2, K73.8, K73.9 for chronic hepatitis; K74.3 and K74.5 for biliary cirrhosis; and K75.4 for autoimmune hepatitis.

### 2.2. Laboratory and Clinical Data

All blood samples were collected in the early morning hours following an overnight fast of at least 8 h as part of routine clinical assessments upon hospital admission. Laboratory testing was conducted in the same certified central laboratory using standardized, automated analyzers and protocols that adhered to internal and external quality control regulations. This ensured consistency in the measurement of hematological, biochemical, and coagulation parameters across all patients included in the study. Reagents, calibration methods, and analyzer models remained unchanged throughout the study period to maintain uniformity in data acquisition. Blood samples were obtained during routine clinical evaluations, and a series of tests were performed. The complete blood count (CBC) included white blood cell count, differential leukocyte count, red blood cell count, hemoglobin levels, hematocrit, and platelet count, which were subsequently used to compute various hemogram-derived ratios. Liver function tests encompassed the measurement of aspartate aminotransferase (AST), alanine aminotransferase (ALT), alkaline phosphatase (ALP), and gamma-glutamyl transferase (GGT) using automated biochemical analyzers. Albumin levels were also recorded as part of liver function assessment. These enzyme levels were crucial for calculating hepatic steatosis and fibrosis scores.

The coagulation profile was also analyzed, including prothrombin time (PT), activated partial thromboplastin time (APTT), fibrinogen levels, and international normalized ratio (INR). Additionally, the lipid profile, including low-density lipoprotein (LDL), high-density lipoprotein (HDL), total cholesterol, and triglycerides, was assessed to evaluate metabolic status and support the computation of specific hepatic indices.

Furthermore, the Child–Pugh criteria were applied to classify the severity of liver disease by incorporating both clinical and laboratory parameters. The Child–Pugh score includes five factors: bilirubin level, albumin level, INR, presence and severity of ascites, and degree of hepatic encephalopathy [11]. Each parameter was scored from 1 to 3, with a total score categorizing patients into class A (5–6 points, well-compensated disease), class B (7–9 points, significant functional compromise), or class C (10–15 points, decompensated disease). Also, the MELD score was calculated, and associated mortality risk was evaluated [11,12].

Renal function was determined by measuring creatinine and urea levels, with the estimated glomerular filtration rate (eGFR) calculated accordingly. Fasting plasma glucose levels were also recorded to identify metabolic abnormalities potentially linked to hepatic steatosis.

### 2.3. Hepatic Steatosis and Liver Fibrosis Scores

The assessment of hepatic steatosis and liver fibrosis was conducted using a range of non-invasive scoring systems, as outlined in Table 1. These included the fibrosis-4 index (FIB-4), AST to platelet ratio index (APRI), NAFLD fibrosis score (NFS), and the aspartate aminotransferase to alanine aminotransferase (AST/ALT) ratio. Additionally, other predictive models, such as the body mass index, AST/ALT ratio, and diabetes (BARD) score; the age, bilirubin, INR, and serum creatinine level (ABIC) score; King’s score for liver fibrosis; the logarithm of odds for Lok index (LogOddsLok); Lok index for liver fibrosis (Lok index); the triglyceride to glucose (TyG) ratio; prognostic nutritional index (PNI); platelet–albumin–bilirubin (PALBI) score; albumin–bilirubin (ALBI) score; and the fibrosis index were utilized [13,14,15,16]. These indices were derived from routine laboratory test results and provided insight into the extent of hepatic impairment within the study cohort. The probability of advanced fibrosis was determined in accordance with established guidelines specific to each scoring method

### 2.4. Hemogram-Derived Ratios and Biomarkers

Various hemogram-derived ratios and systemic inflammatory markers were evaluated to further characterize the inflammatory status of patients. These included the neutrophil-to-lymphocyte ratio (NLR), derived neutrophil-to-lymphocyte ratio (dNLR), platelet-to-lymphocyte ratio (PLR), platelet-to-neutrophil ratio (PNR), lymphocyte-to-monocyte ratio (LMR), eosinophil-to-lymphocyte ratio (ELR), basophil-to-lymphocyte ratio (BLR), neutrophil–lymphocyte–albumin ratio (NLAR), and advanced inflammatory score index (AISI) [17]. Additionally, the red cell distribution width (RDW-CV)-to-platelet ratio and the systemic immune–inflammation index (SII), which is calculated as (platelet count × neutrophil count)/lymphocyte count, were analyzed.

### 2.5. Statistical Analysis

Descriptive statistics were used to summarize the data, with normally distributed quantitative variables presented as means and standard deviations (SDs) and non-normally distributed data expressed as medians with interquartile ranges (IQRs). Categorical variables were reported as frequencies and percentages. To compare clinical characteristics between groups, appropriate statistical tests were applied: the independent *t*-test for normally distributed quantitative variables, the Wilcoxon rank-sum test for non-normally distributed quantitative data, and either the chi-square (χ^2^) test or Fisher’s exact test for categorical variables. The receiver operating characteristic (ROC) curve analysis was conducted to evaluate the performance of hepatic steatosis and liver fibrosis scores, along with hemogram-derived ratios, in distinguishing between CLD and cirrhosis. A *p*-value of <0.05 was considered statistically significant. All statistical analyses were performed using R software version 4.1.2 (R Foundation for Statistical Computing, Vienna, Austria).

## 3. Results

### 3.1. General Characteristics

The studied population included a total of 250 individuals, with a median age of 64 years (IQR: 56–69), ranging from 19 to 89 years. Of the 250 participants, 113 had CLD without cirrhosis (45.2%), and 137 had liver cirrhosis (54.8%). A significant difference was observed in the sex distribution, with a higher proportion of females in the CLD group (72.57%) compared to the cirrhosis group (34.31%) (*p*-value < 0.001). Hypertension was more common in CLD patients (61.95%) than in cirrhosis patients (47.45%) (*p*-value = 0.022), while diabetes was more prevalent in the cirrhosis group (31.39%) compared to the CLD group (16.81%) (*p*-value = 0.008). Dyslipidemia and statin use were significantly higher in the CLD compared to the cirrhosis group (*p*-value < 0.001). The proportion of individuals with metabolic syndrome was higher in CLD patients compared to cirrhosis patients, but the difference was not statistically significant (*p*-value = 0.084). Body mass index (BMI) did not differ significantly between the two groups (*p*-value = 0.539). Table 2 summarizes the general characteristics of the included participants.

### 3.2. CLD and Cirrhosis Etiologies

The etiologies of CLD and liver cirrhosis showed notable differences, as outlined in Table 3. In the CLD group, the most common causes were hepatitis B (37.17%), hepatitis C (34.51%), and autoimmune hepatitis (14.16%), followed by primary biliary cholangitis (12.39%). In contrast, liver cirrhosis was predominantly associated with alcohol-related liver disease (46.72%), followed by hepatitis C (24.09%), hepatitis B (10.95%), and hepatitis B and C co-infection (2.19%). Other causes of cirrhosis included autoimmune overlap (2.92%), cryptogenic or Wilson’s disease (6.57%), and primary biliary cirrhosis (0.73%). The difference in the distribution of etiologies between the two groups was statistically significant (*p*-value < 0.001).

### 3.3. Laboratory Tests

The laboratory blood tests showed significant differences between the CLD and cirrhosis groups, as reported in Table 4. Hemoglobin, hematocrit, and platelet counts were significantly lower in the cirrhosis group (*p*-value < 0.001). Lymphocyte count was also significantly reduced in the cirrhosis group (*p*-value < 0.001). In metabolic variables, glycemia, triglycerides, LDL, and HDL levels showed significant differences, with higher glycemia and triglycerides and lower LDL and HDL in the cirrhosis group (*p*-value = 0.011, *p*-value = 0.007, *p*-value < 0.001, *p*-value < 0.001, respectively). Liver function tests indicated significantly lower albumin and significantly higher ASAT, GGT, alkaline phosphatase, and total bilirubin levels in the cirrhosis group (*p*-value < 0.001 for all). The INR was also significantly higher in the cirrhosis group (*p*-value < 0.001).

### 3.4. Clinical Features and Prognostic Scores

Encephalopathy and ascites were significantly more common in the cirrhosis group, as demonstrated in Table 5. The Child–Pugh classification showed that cirrhosis patients were distributed across class A (41.6%), class B (38.4%), and class C (20%) (*p*-value < 0.001). In terms of ALBI grade, the majority of CLD patients were in grade A2, while cirrhosis patients were predominantly in grade A3 (*p*-value < 0.001). ALBI scores and MELD scores were significantly higher in the cirrhosis group, indicating greater liver dysfunction (*p*-value < 0.001). Furthermore, mortality risk was substantially higher in the cirrhosis group compared to the CLD group (*p*-value < 0.001).

### 3.5. Non-Invasive Biomarkers and Scores in Chronic Liver Disease and Liver Cirrhosis

Significant differences were found between CLD and cirrhosis patients in several biomarkers and scores, as outlined in Table 6, including APRI (*p*-value < 0.001), FIB-4 (*p*-value < 0.001), ASAT/ALAT ratio (*p*-value < 0.001), and NFS (*p*-value < 0.001), with cirrhosis patients showing higher values, indicating more advanced liver disease. The prognostic nutritional index (*p*-value < 0.001) was lower in cirrhosis patients, reflecting poorer nutritional status. The PALBI score showed no significant difference between groups (*p*-value = 0.44). Other scores, such as the ABIC, fibrosis index, King score, and Lok index, also had significantly higher values in the cirrhosis group, indicating greater liver damage and severity in comparison to the CLD group (*p*-value < 0.001).

### 3.6. Hemogram-Derived Ratios

In this comparison of hemogram-derived ratios between patients with CLD and liver cirrhosis, several significant differences were observed, as reported in Table 7. The NLR was higher in cirrhosis patients (*p*-value < 0.001), while the PLR was significantly lower in cirrhosis patients compared to those with CLD (*p*-value = 0.002). Other ratios, including the LMR, PNR, and neutrophil–lymphocyte–albumin ratio, showed significant differences (*p*-value < 0.001), indicating more systemic inflammation in cirrhosis. Conversely, no significant differences were observed for the dNLR, SII, AISI, ELR, and BLR between the two groups (*p*-value > 0.05).

### 3.7. Predictive Accuracy of Several Biomarkers and Scores in Differentiating CLD from Cirrhosis

**Figure 1:**
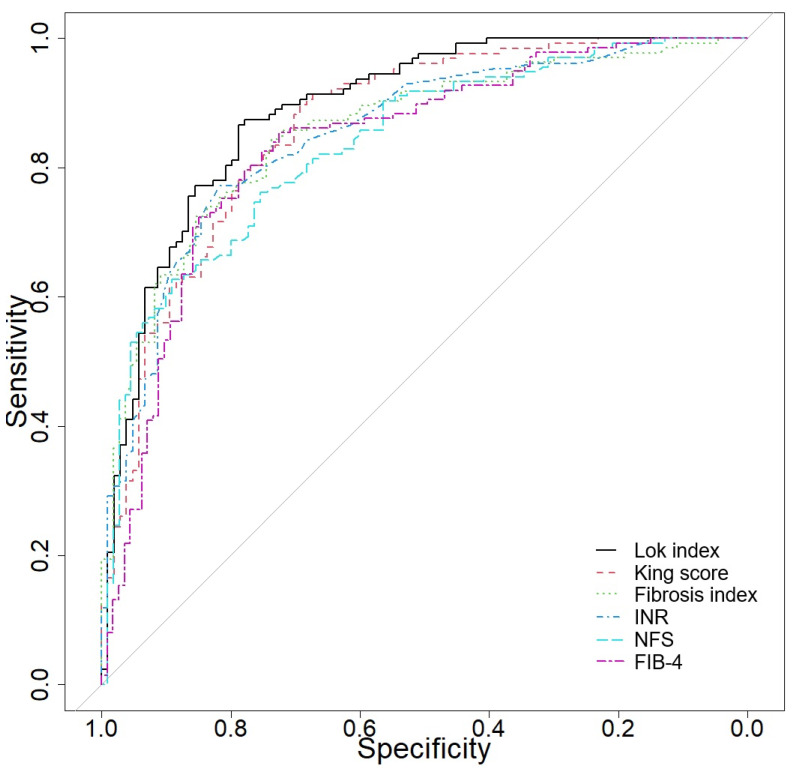
Receiver operator characteristic curve in differentiating CLD vs. cirrhosis.

Table 8 and Figure 1 summarize the performance of various clinical variables in distinguishing between cirrhosis and other CLD based on their AUC, sensitivity (Se), specificity (Sp), and optimal cut-off values. The Lok index demonstrated the highest AUC of 0.89, with a sensitivity of 86.61% and specificity of 78.85%. Other key variables, such as the King score (AUC 0.864), fibrosis index (AUC 0.856), INR (AUC 0.851), and NFS (AUC 0.836), also displayed excellent discriminatory power with high sensitivities and specificities. Conversely, variables like the PALBI score (AUC 0.529), BLR (AUC 0.526), and AISI (AUC 0.513) showed lower diagnostic accuracy.

## 4. Discussion

CLD continues to represent a major global health concern due to its silent progression and high risk of complications, including cirrhosis and hepatocellular carcinoma [18,19]. The ability to accurately assess disease severity and predict progression using non-invasive biomarkers and scoring systems is critical in optimizing patient management [20]. In this study, we investigated the utility of various biomarkers, scores, and hemogram-derived ratios in differentiating CLD from cirrhosis, as well as their predictive accuracy. Our findings highlight significant differences in laboratory parameters, hemogram-derived ratios, and established non-invasive fibrosis scores between CLD and cirrhosis patients, underscoring their potential clinical utility in disease stratification.

The observed hematological and biochemical alterations in cirrhosis align with previous studies, highlighting anemia and thrombocytopenia due to hypersplenism and bone marrow suppression [21]. Reduced lymphocyte count supports the known immune dysfunction in cirrhosis. Metabolic disturbances, including altered lipid profiles and elevated glycemia, reflect impaired hepatic synthesis and systemic inflammation. Abnormal liver function tests and increased INR confirm hepatic dysfunction and coagulopathy, consistent with existing literature [21,22]. These findings reinforce the systemic impact of cirrhosis and the utility of routine blood tests in assessing disease severity.

Our findings demonstrate the utility of non-invasive biomarkers and fibrosis scores in differentiating between CLD and cirrhosis. The well-established indices, such as APRI and FIB-4, have been widely validated as reliable tools for assessing liver fibrosis, with prior research confirming their strong correlation with histological staging [5,23]. Similarly, the ASAT/ALAT ratio and NFS have been recognized as valuable indicators of liver disease progression, consistent with our observations [24]. The lower prognostic nutritional index in cirrhosis patients supports existing evidence highlighting the role of malnutrition in advanced liver disease, emphasizing its prognostic significance. While the PALBI score has been reported as a predictor of hepatic decompensation and survival in cirrhosis [25], its lack of discrimination between CLD and cirrhosis in our cohort may reflect variations in patient characteristics or differences in disease severity. Additionally, fibrosis-related markers such as the ABIC, fibrosis index, King score, and Lok index have been previously shown to correlate with liver fibrosis progression, reinforcing their relevance in staging CLD [10,26,27]. These findings support the broader literature advocating for non-invasive markers as alternatives to liver biopsy, although further validation is necessary to refine their clinical applicability and establish standardized cut-off values across diverse populations.

Our study identified distinct cut-off values for non-invasive fibrosis scores when differentiating CLD from cirrhosis, which varied compared to other published studies that included different etiologies. These findings have important clinical implications, particularly in tailoring non-invasive diagnostic strategies to specific patient populations. The recalibrated cut-offs offer clinicians an approach to interpreting fibrosis scores, helping avoid misclassification that could arise from applying thresholds validated predominantly in MASLD or other etiology cohorts. For example, using a lower Lok index cut-off of 0.63 in this setting may improve early cirrhosis detection in viral or autoimmune liver disease, thereby prompting timely intervention and monitoring. For instance, the optimal APRI cut-off in our cohort was 0.638, slightly higher than the values reported in MASLD populations [28]. Similarly, our FIB-4 cut-off of 2.19 was higher than those reported in MASLD studies, where values above 2.67–3.25 are more predictive of advanced fibrosis [28]. The Lok index demonstrated the highest accuracy in our study, with a cut-off of 0.63, which is in line with prior studies in viral hepatitis [29]. Additionally, the King score and fibrosis index showed strong discriminatory power with cut-offs of 11.40 and 2.19, respectively, slightly higher than those reported in mixed-etiology studies, possibly due to differences in fibrosis progression patterns [30]. Interestingly, our NFS cut-off of 0.53 was higher than those seen in non-alcoholic fatty liver disease (NAFLD) studies, where negative values (e.g., <−1.455) typically exclude advanced fibrosis [31]. Although the Lok index, King score, and FIB-4 have been previously validated in large, mixed-etiology populations, the cut-off values established in those studies may not be directly applicable to narrower clinical cohorts. Our study focused specifically on patients with viral, autoimmune, or cholestatic liver disease, excluding MASLD and ALD, where fibrosis progression patterns and inflammatory profiles differ. The ROC analysis in our cohort identified optimized cut-off points that vary from traditionally used thresholds, supporting the need for context-specific recalibration. These adjusted cut-offs can enhance diagnostic precision in targeted populations by minimizing the risk of over- or underestimating fibrosis severity, and they underscore the impact of underlying etiology on score performance, highlighting the need for etiology-specific thresholds to improve clinical accuracy and applicability.

The hemogram-derived ratios evaluated in our study provide valuable insights into systemic inflammation and liver disease severity. Although the discriminatory power of NLR and PLR was modest, their significant differences between groups suggest a potential adjunctive role in the broader assessment of liver disease severity. Given their ease of calculation from routine blood tests, these ratios could complement other markers in identifying patients who may warrant further fibrosis evaluation, especially in resource-limited settings. However, their use should be contextualized within a multimodal diagnostic approach rather than as standalone tools. Our findings align with previous research demonstrating that elevated NLR in cirrhosis reflects heightened systemic inflammation and immune dysregulation [10,32]. The lower PLR in cirrhosis may be linked to thrombocytopenia and altered platelet function, as reported in prior studies [33]. Significant differences in LMR, PNR, and neutrophil–lymphocyte–albumin ratio further support their potential as inflammatory markers in advanced liver disease [10]. However, the lack of significant differences in dNLR, SII, AISI, ELR, and BLR suggests these indices may be less sensitive in distinguishing cirrhosis from CLD. Nevertheless, their potential role in liver disease assessment warrants further investigation.

Importantly, while hemogram-derived ratios such as NLR, PLR, LMR, and PNR showed statistically significant group differences, their overall diagnostic performance was limited. This finding adds to the growing body of literature questioning the reliability of inflammation-based indices in fibrosis staging. It also emphasizes the need to distinguish statistical significance from clinical utility, particularly when biomarkers are derived from highly variable components like white blood cell differentials. Future studies should explore whether integrating these ratios into composite indices or using dynamic rather than static measurements could enhance their diagnostic contribution.

Our findings highlight the strong discriminatory power of non-invasive fibrosis scores such as the Lok index, King score, fibrosis index, INR, and NFS, which demonstrated high AUC values, emphasizing their reliability in identifying cirrhosis. The FIB-4 index, MELD score, and RDW-to-platelet ratio also showed good diagnostic accuracy, reinforcing their clinical relevance in liver disease staging [10,34]. In contrast, markers like PALBI, BLR, and AISI had poor diagnostic performance, suggesting their limited role in fibrosis assessment. Similarly, hematologic indices such as SII, ELR, and dNLR exhibited weak discriminatory power, indicating their potential inefficacy in differentiating cirrhosis from CLD. These findings underscore the importance of selecting appropriate non-invasive markers for fibrosis evaluation while encouraging future research to refine predictive models that integrate multiple biomarkers for improved diagnostic accuracy. Collectively, these findings may support a more nuanced, etiology-aware application of non-invasive fibrosis scores and inflammatory indices in clinical hepatology. By refining existing diagnostic thresholds and exploring additional blood-based markers, this work contributes to a more personalized and accessible approach to CLD assessment. Several limitations must be acknowledged in our study. First, its retrospective design inherently limits causal inferences, and the findings should be interpreted within this context. Additionally, the study was conducted in a single tertiary care center, which may limit the generalizability of the results to broader populations. Moreover, while we assessed a wide array of biomarkers and scoring systems, the lack of long-term follow-up data prevents an evaluation of their prognostic significance in predicting clinical outcomes such as decompensation, transplantation, or mortality. Our study did not incorporate imaging techniques such as elastography or MRI-based methods for the evaluation of liver fibrosis, which could provide additional diagnostic accuracy. Furthermore, we excluded metabolic and alcohol-related etiologies in CLD groups but included all etiologies of liver cirrhosis. This is mainly because we cannot evaluate retrospectively whether a patient has MASLD, ALD, or MetALD. Hence, in such situations, results might differ significantly and could introduce bias due to incorrect grouping. We identified distinct cut-off values for several non-invasive fibrosis scores, which differed from those reported in mixed-etiology cohorts. This likely reflects variation in fibrosis progression across disease types. Notably, while the CLD group included only viral, autoimmune, or cholestatic etiologies, the cirrhosis group encompassed all causes, including metabolic and alcohol-related diseases. This introduces a methodological limitation that constrains direct etiological comparisons. As a result, the observed differences in score performance should be interpreted with caution. Finally, notable demographic and clinical differences between the CLD and cirrhosis groups, such as disparities in sex distribution and prevalence of comorbidities like hypertension, diabetes, and dyslipidemia, may have acted as confounding variables. These differences likely stem from the distinct etiological patterns of liver disease within each group (e.g., higher viral and autoimmune etiologies in CLD vs. alcohol-related causes in cirrhosis). While they reflect the natural heterogeneity of liver disease in clinical settings, these baseline imbalances may influence biomarker levels and introduce bias. Future prospective studies with matched cohorts are warranted to minimize the impact of such confounding factors and enhance internal validity.

Despite these limitations, our study has notable strengths. It provides a comprehensive evaluation of both conventional and emerging non-invasive biomarkers for liver disease assessment. The inclusion of hemogram-derived ratios expands on existing knowledge, emphasizing their potential role in distinguishing CLD from cirrhosis. Furthermore, the use of a robust statistical methodology, including ROC curve analysis, enhances the validity of our findings. By reinforcing the utility of non-invasive diagnostic approaches, our study contributes to the ongoing efforts to refine CLD management and minimize the reliance on invasive procedures such as liver biopsy. Future prospective studies with larger cohorts and longitudinal follow-up are needed to validate these findings and further elucidate the predictive value of these biomarkers in liver disease progression.

## 5. Conclusions

In conclusion, our study highlights the potential utility of several non-invasive scores in differentiating CLD from liver cirrhosis. We confirmed that non-invasive fibrosis scores such as the Lok index, King score, fibrosis index, and NFS remain reliable tools for disease stratification. These tools can be incorporated into clinical practice to assist in early detection and risk stratification, potentially reducing the need for invasive procedures like liver biopsy. Moreover, although revealing statistically significant differences between CLD and cirrhosis, hemogram-derived ratios demonstrated limited diagnostic performance and should be incorporated into a multimodal diagnostic approach rather than used as standalone markers. Clinicians should use these markers as part of a broader diagnostic framework, ensuring more accurate and individualized management of liver disease.

## Figures and Tables

**Table 1:** Formulas for several non-invasive biomarkers and scores.

Score	Formula
Fibrosis-4 Index (FIB-4)	Age (years) × AST (U/L)/{platelet (×10^9^/L) × [ALT (U/L)]^1/2^}
AST to Platelet Ratio Index (APRI)	[(AST/ULN AST)/platelet (×10^9^/L)] × 100
NAFLD Fibrosis Score (NFS)	−1.675 + (0.037 × Age) + (0.094 × BMI) + (1.13 × IFG/DM) + (0.99 × AST/ALT) − (0.013 × Platelets) − (0.66 × Albumin)
AST/ALT Ratio	AST/ALT
BARD Score	BMI ≥ 28 = 1 point, AST/ALT ≥ 0.8 = 2 points, Diabetes = 1 point
ABIC Score	(0.62 × Age) + (0.91 × Bilirubin) + (0.48 × INR) + (0.11 × Creatinine)
King’s Score	Age (years) × AST (U/L) × INR/Platelet (10^9^/L)
LogOddsLok	(1.26 × AST/ALT) + (5.27 × INR) − [0.0089 × Platelet (×10^9^/L)] − 5.56
Lok Index	e^(LogOddsLok)^/(1 + e^(LogOddsLok)^)
Triglyceride–Glucose (TyG) Ratio	Fasting Triglycerides × Fasting Glucose/2
Prognostic Nutritional Index (PNI)	(10 × Albumin [g/dL]) + (0.005 × Total Lymphocyte Count)
MELD score	9.57 × loge (creatinine) + 3.78 × loge (total bilirubin) + 11.2 × loge (INR) + 6.43
Platelet-Albumin-Bilirubin (PALBI) Score	2.02 × log_10_[bilirubin (μmol/L)] − 0.37 × [log_10_(bilirubin)]^2^ − 0.04 × albumin (g/L) − 3.48 × log_10_[platelets (×10^9^/L)] + 1.01 × log_10_(platelets)
Albumin-Bilirubin (ALBI) Score	[log_10_(bilirubin (μmol/L)] × 0.66 − [Albumin (g/L) × 0.085]
ALBI grade	ALBI grade 1 (≤−2.60) ALBI grade 2 (>−2.60 to ≤−1.39) ALBI grade 3 (>−1.39)
Fibrosis Index	8 − 0.01 × platelets (109/L) − albumin (g/dL)

ABIC—age, bilirubin, INR, and creatinine score; ALBI—albumin–bilirubin; ALT—alanine aminotransferase; APRI—AST to platelet ratio index; AST—aspartate aminotransferase; BARD—BMI, AST/ALT ratio, diabetes; BMI—body mass index; DM—diabetes mellitus; FIB-4—fibrosis-4 index; IFG—impaired fasting glucose; INR—international normalized ratio; LogOddsLok—logarithm of odds for Lok index; MELD—model for end-stage liver disease; NAFLD—non-alcoholic fatty liver disease; NFS—NAFLD fibrosis score; PALBI—platelet–albumin–bilirubin; PNI—prognostic nutritional index; TyG—triglyceride–glucose ratio; ULN—upper limit of normal.

**Table 2:** General characteristics of the included participants.

Variable	CLD (*n* = 113)	Cirrhosis (*n* = 137)	*p*-Value
Sex (F), nr (%)	82 (72.57)	47 (34.31)	<0.001
Age (years), median (IQR)	63 (53–69)	64 (59–70)	0.052
BMI (kg/m^2^), median (IQR)	22.49 (21–32)	21 (21–31.25)	0.539
Sex (F), nr (%)	82 (72.57)	47 (34.31)	<0.001
Hypertension, *n* (%)	70 (61.95)	65 (47.45)	0.022
Diabetes, *n* (%)	19 (16.81)	43 (31.39)	0.008
Insulin use, *n* (%)	11 (9.73)	23 (16.79)	0.105
Dyslipidemia, *n* (%)	43 (38.05)	12 (8.76)	<0.001
Statin use, *n* (%)	43 (38.05)	12 (8.76)	<0.001
Metabolic syndrome, *n* (%)	30 (26.55)	24 (17.52)	0.084

**Table 3:** Different etiologies in CLD and cirrhosis patients.

Etiologies	CLD (*n* = 113)	Cirrhosis (*n* = 137)	*p*-Value
Hepatitis B, nr (%)	42 (37.17)	15 (10.95)	<0.001
Hepatitis C, nr (%)	39 (34.51)	33 (24.09)	
Hepatitis B–C co-infection, nr (%)	1 (0.88)	3 (2.19)	
Hepatitis B–D co-infection, nr (%)	1 (0.88)	0 (0)	
Primary biliary cholangitis, nr (%)	14 (12.39)	1 (0.73)	
Autoimmune hepatitis, nr (%)	16 (14.16)	8 (5.84)	
Alcohol-related, nr (%)	0 (0)	64 (46.72)	
Overlap (autoimmune + PBC), nr (%)	0 (0)	4 (2.92)	
Other causes (Cryptogenic, Wilson’s disease, Drug induced), nr (%)	0 (0)	9 (6.57)	

**Table 4:** Laboratory blood tests of included participants.

Variable	CLD (*n* = 113)	Cirrhosis (*n* = 137)	*p*-Value
Complete blood count			
Hemoglobin (g/dL), median (IQR)	13.2 (12.4–14.5)	11.8 (10–13.5)	<0.001
Hematocrit (%), median (IQR)	39.3 (37–42.4)	34.5 (30.1–39.7)	<0.001
Leukocytes/L, median (IQR)	6.12 (5.02–8.09)	6.48 (4.3–9.35)	0.838
Platelets/L, median (IQR)	217 (177–265)	122 (84–179)	<0.001
Neutrophils × 10^9^/L, median (IQR)	3.8 (2.88–4.9)	3.99 (2.62–6.28)	0.582
Lymphocytes × 10^9^/L, median (IQR)	1.68 (1.3–2.21)	1.26 (0.74–1.63)	<0.001
Leucocytes × 10^9^/L, median (IQR)	6.12 (5.02–8.09)	6.48 (4.3–9.35)	0.838
Eosinophils × 10^9^/L, median (IQR)	0.12 (0.06–0.19)	0.09 (0.05–0.17)	0.224
Monocytes × 10^9^/L, median (IQR)	0.43 (0.31–0.51)	0.45 (0.32–0.66)	0.104
Basophils × 10^9^/L, median (IQR)	0.03 (0.02–0.04)	0.03 (0.01–0.04)	0.105
RDW-CV (%), median (IQR)	13.45 (12.9–14.33)	14.6 (13.6–16.4)	<0.001
PDW (fL), median (IQR)	16.2 (15.95–16.45)	16.3 (16.1–16.65)	0.009
Metabolic blood tests			
Glycemia, median (IQR)	101 (92–117)	110 (95–137)	0.011
Triglycerides, median (IQR)	103.5 (85.75–139.75)	88.5 (72.75–125.75)	0.007
LDL, median (IQR)	110.5 (87.5–134)	71 (49–94)	<0.001
HDL, median (IQR)	49 (40–59)	40 (28–54)	<0.001
Liver function tests			
Albumin (g/dL), median (IQR)	4.2 (3.9–4.5)	3.37 (2.8–4)	<0.001
Creatinine (mg/dL), median (IQR)	0.75 (0.66–0.98)	0.85 (0.66–1.2)	0.055
ASAT (U/L), median (IQR)	28 (22–40)	45 (29–71)	<0.001
ALAT (U/L), median (IQR)	25 (16–39)	26 (17–38)	0.516
GGT (U/L), median (IQR)	31 (18–77)	85 (34.25–168)	<0.001
Alkaline Phosphatase, median (IQR)	72 (57–97)	96 (72.5–143)	<0.001
Total Bilirubin mg/dL), median (IQR)	0.65 (0.5–0.93)	1.39 (0.84–2.54)	<0.001
Coagulation profile			
INR (sec), median (IQR)	1.01 (0.96–1.09)	1.28 (1.12–1.62)	<0.001

ALAT—alanine aminotransferase; ASAT—aspartate aminotransferase; CLD—chronic liver disease; GGT—gamma-glutamyl transferase; HDL—high-density lipoprotein; INR—international normalized ratio; LDL—low-density lipoprotein; PDW—platelet distribution width; RDW-CV—red cell distribution width coefficient of variation.

**Table 5:** Clinical features and prognostic scores.

Variable	CLD (*n* = 113)	Cirrhosis (*n* = 137)	*p*-Value
Encephalopathy, *n* (%)	absent: 113 (100)	absent: 104 (75.91)	<0.001
	moderate: 0 (0)	moderate: 25 (18.25)	
	severe: 0 (0)	severe: 8 (5.84)	
Ascites, *n* (%)	absent: 110 (97.35)	absent: 77 (56.2)	<0.001
	moderate: 3 (2.65)	moderate: 37 (27.01)	
	severe: 0 (0)	severe: 23 (16.79)	
Child–Pugh Class, *n* (%)	A: 113 (100)	A: 52 (41.6)	<0.001
	B: 0 (0)	B: 48 (38.4)	
	C: 0 (0)	C: 25 (20)	
ALBI grade, *n* (%)	A1: 7 (6.42)	A1: 2 (1.5)	<0.001
	A2: 90 (82.57)	A2: 44 (33.08)	
	A3: 12 (11.01)	A3: 87 (65.41)	
ALBI score, median (IQR)	−1.91 (−2.3–−1.63)	−0.87 (−1.64–−0.27)	<0.001
MELD, median (IQR)	2.51 (0.46–5.94)	9.34 (5.02–14.74)	<0.001
Mortality risk %, median (IQR)	1.9 (1.9–1.9)	6 (1.9–6)	<0.001

ALBI—albumin–bilirubin; Child–Pugh Class—Child–Turcotte–Pugh Classification; MELD—model for end-stage liver disease.

**Table 6:** Non-invasive biomarkers and scores in CLD and cirrhosis patients.

Variable	CLD (*n* = 113)	Cirrhosis (*n* = 137)	*p*-Value
APRI, median (IQR)	0.37 (0.27–0.55)	1.1 (0.57–2.27)	<0.001
FIB4, median (IQR)	1.68 (1.15–2.28)	4.89 (2.79–9.31)	<0.001
NFS, median (IQR)	−1.31 (−2.24–−0.05)	1.14 (0–2.21)	<0.001
Triglyceride–glucose index, median (IQR)	3.76 (3.61–3.91)	3.71 (3.58–3.88)	0.426
Prognostic nutritional index, median (IQR)	42.82 (39.46–45.81)	34.43 (29.1–40.71)	<0.001
AST/ALT ratio, median (IQR)	1.14 (0.91–1.5)	1.77 (1.33–2.29)	<0.001
AST to platelet ratio index (APRI), median (IQR)	0.37 (0.27–0.55)	1.1 (0.57–2.27)	<0.001
Platelet–albumin–bilirubin (PALBI) score, median (IQR)	−3.86 (−3.97–−3.68)	−3.86 (−3.97–−3.76)	0.44
ABIC, median (IQR)	7.31 (6.4–8.05)	8.05 (7.52–8.83)	<0.001
Fibrosis index, median (IQR)	1.65 (1.02–2.34)	3.44 (2.49–3.95)	<0.001
King score, median (IQR)	7.39 (5.16–13.04)	36.67 (15.67–74.59)	<0.001
Lok index, median (IQR)	0.34 (0.24–0.57)	0.92 (0.78–0.99)	<0.001

ABIC—age, bilirubin, INR, and serum creatinine level; APRI—AST to platelet ratio index; AST/ALT—aspartate aminotransferase to alanine aminotransferase ratio; FIB4—fibrosis-4 index; NFS—NAFLD fibrosis score; PALBI—platelet–albumin–bilirubin score.

**Table 7:** Hemogram derived ratios in CLD and cirrhosis patients.

Variable	CLD (*n* = 113)	Cirrhosis (*n* = 137)	*p*-Value
NLR, median (IQR)	2.18 (1.7–3.3)	3.2 (2.2–5.65)	<0.001
dNLR, median (IQR)	1.6 (1.21–2.24)	1.68 (1.06–2.49)	0.634
PLR, median (IQR)	129.32 (92.06–173.14)	104.46 (59.61–157.68)	0.002
LMR, median (IQR)	4.06 (3.3–5.42)	2.74 (1.65–3.94)	<0.001
PNR, median (IQR)	55.64 (39.32–68.83)	29.36 (20.37–45.21)	<0.001
SII, median (IQR)	439.46 (328.98–701.47)	393.19 (213.5–763.19)	0.109
AISI, median (IQR)	191.2 (113.2–313.71)	189.14 (73.02–465.46)	0.735
ELR, median (IQR)	0.07 (0.04–0.11)	0.09 (0.05–0.15)	0.098
BLR, median (IQR)	0.02 (0.01–0.03)	0.02 (0.01–0.03)	0.486
Neutrophil–lymphocyte–albumin ratio, median (IQR)	0.51 (0.4–0.77)	0.98 (0.55–2.03)	<0.001

AISI—aggregate index of systemic inflammation; BLR—basophil-to-lymphocyte ratio; dNLR—derived neutrophil-to-lymphocyte ratio; ELR—eosinophil-to-lymphocyte ratio; LMR—lymphocyte-to-monocyte ratio; NLR—neutrophil-to-lymphocyte ratio; PNR—platelet-to-neutrophil ratio; PLR—platelet-to-lymphocyte ratio; SII—systemic immune–inflammation index.

**Table 8:** Receiver operator characteristics in differentiating CLD vs. cirrhosis.

Variable	AUC (95% CI)	Se	Sp	Cut-Off
Lok index	0.89 (0.848–0.93)	86.61	78.85	0.633044469
King score	0.864 (0.812–0.91)	89.76	69.23	11.40065789
Fibrosis index	0.856 (0.807–0.902)	84.33	73.64	2.19
INR (sec)	0.851 (0.8–0.899)	77.17	81.73	1.11
NFS	0.836 (0.785–0.881)	62.69	89.09	0.53
FIB-4-index	0.834 (0.782–0.885)	85.4	72.57	2.186795399
Score 5	0.83 (0.787–0.875)	72	87	5
RDW-to-platelet ratio	0.826 (0.77–0.876)	82.96	73.21	0.07
ALBI score	0.823 (0.773–0.872)	71.43	85.32	−1.508713864
APRI	0.805 (0.749–0.859)	70.07	83.19	0.638297872
MELD	0.802 (0.743–0.855)	79.37	67.65	4.51
Platelets × 10^9^/L	0.795 (0.737–0.848)	79.65	72.26	169
Prognostic nutritional index	0.776 (0.718–0.833)	89.91	59.09	35.64
Albumin (g/dL)	0.775 (0.711–0.83)	90	58.96	3.52
PNR	0.767 (0.707–0.825)	64.29	81.48	48.46
AST/ALT ratio	0.748 (0.684–0.806)	70.8	73.45	1.428571429
LMR	0.72 (0.654–0.778)	74.11	66.67	3.37
Neutrophil–lymphocyte–albumin ratio	0.708 (0.641–0.773)	60.61	76.15	0.79
ABIC	0.707 (0.638–0.773)	73.81	61.76	7.5362
RDW-CV (%)	0.706 (0.639–0.769)	73.33	59.82	13.6
Lymphocytes × 10^9^/L	0.685 (0.618–0.748)	69.64	61.48	1.39
NLR	0.658 (0.586–0.726)	73.33	54.46	2.23
PLR	0.615 (0.548–0.683)	93.75	34.07	69.88
PDW (fL)	0.596 (0.528–0.666)	59.26	56.76	16.2
Creatinine (mg/dL)	0.571 (0.499–0.64)	61.31	57.14	0.77
ELR	0.561 (0.489–0.631)	48.15	66.07	0.09047619
Monocytes × 10^9^/L	0.56 (0.486–0.631)	34.07	84.82	0.56
SII	0.559 (0.487–0.634)	91.96	27.41	230.53
Eosinophils × 10^9^/L	0.545 (0.471–0.616)	60.71	54.07	0.1
Triglyceride–glucose index	0.531 (0.457–0.607)	45.71	66.67	3.791374811
Platelet–albumin–bilirubin (PALBI) score	0.529 (0.458–0.603)	24.77	85.71	−3.675684415
BLR	0.526 (0.455–0.596)	25.19	88.39	0.032786885
Neutrophils × 10^9^/L	0.52 (0.449–0.591)	34.07	79.46	5.28
dNLR	0.518 (0.447–0.589)	49.63	59.82	1.72
AISI	0.513 (0.438–0.586)	95.54	24.44	64.95828571
Leucocytes × 10^9^/L	0.508 (0.435–0.581)	27.01	86.73	9.23
Basophils × 10^9^/L	0.441 (0.371–0.513)	61.61	48.89	0.02

ABIC—age, bilirubin, INR, and serum creatinine level; AISI—aggregate index of systemic inflammation; ALT—alanine aminotransferase; AST—aspartate aminotransferase; APRI—AST to platelet ratio index; AUC—area under the curve; BLR—basophil-to-lymphocyte ratio; dNLR—derived neutrophil-to-lymphocyte ratio; ELR—eosinophil-to-lymphocyte ratio; FIB-4—fibrosis-4 index; INR—international normalized ratio; LMR—lymphocyte-to-monocyte ratio; MELD—model for end-stage liver disease; NFS—NAFLD fibrosis score; NLR—neutrophil-to-lymphocyte ratio; PALBI—platelet–albumin–bilirubin score; PLR—platelet-to-lymphocyte ratio; PNR—platelet-to-neutrophil ratio; Se—sensitivity; SII—systemic immune–inflammation index; Sp—specificity.

## Data Availability

The original contributions presented in the study are included in the article; further inquiries can be directed to the corresponding authors.
